# TELEMETRIC-HF: Pilot Randomized Study of Telemetry-Guided Guideline-Directed Medical Therapy Optimization and Ventricular Arrhythmia Surveillance in Patients with Heart Failure with Reduced Ejection Fraction

**DOI:** 10.1159/crd/absag008

**Published:** 2026-08-20

**Authors:** Saša Borović, Dragana Košević, Jelena Stefanović Nešković, Jelena Lešanović, Goran Lončar, Jelena Miladinović, Bojana Milovanović, Petar Dabić, Nadiia Rineiska, Milovan Bojić

**Affiliations:** a National Institute for Heart and Blood Vessels “Dedinje”, Belgrade, Serbia; b University of Belgrade, Faculty of Medicine, Belgrade, Serbia; c State Institute Republican Scientific Practical Centre “Cardiology”, Minsk, Belarus

**Keywords:** Heart failure with reduced ejection fraction, Telemonitoring, Ventricular arrhythmias, Guideline-directed medical therapy, Remote patient management

## Abstract

**Introduction:**

The purpose of the study was to evaluate the feasibility of telemetry-guided optimization of guideline-directed medical therapy (GDMT) and continuous arrhythmic surveillance in ambulatory patients with heart failure with reduced ejection fraction (HFrEF).

**Methods:**

TELEMETRIC-HF was a single-center, randomized, open-label pilot study including 30 patients with HFrEF assigned to telemetry-guided management (*n* = 15) or standard outpatient care (*n* = 15). Patients in the telemetry group underwent 8 weeks of continuous ambulatory monitoring using the CPC12 CE MDR class IIb wearable system, providing continuous electrocardiographic and physiological monitoring with clinician oversight. The primary endpoint was the between-group difference in β-blocker (BB) target-dose achievement at 8 weeks. Secondary endpoints included optimization of other GDMT classes, ventricular arrhythmia detection, clinical status, quality of life, safety, and healthcare utilization.

**Results:**

Telemetry-guided management was associated with significantly greater BB target-dose achievement at 8 weeks than standard care (60.0% vs. 39.2% of the guideline-recommended target dose; absolute difference 20.8%, 95% CI 2.7–39.2; *p* = 0.028). Target-dose achievement for angiotensin receptor-neprilysin inhibitors, mineralocorticoid receptor antagonists, and sodium-glucose cotransporter-2 inhibitors did not differ significantly between groups. Recurrent non-sustained ventricular tachycardia was detected only in the telemetry group, prompting additional clinical evaluation, antiarrhythmic therapy, and primary prevention implantable cardioverter-defibrillator implantation in one patient. Hemodynamic, laboratory, and echocardiographic parameters remained stable throughout follow-up, and telemetry was well tolerated. No heart failure hospitalizations or deaths occurred during the 8-week study period.

**Conclusion:**

In this pilot randomized study, telemetry-guided remote monitoring was feasible and was associated with earlier BB optimization and improved detection of clinically relevant ventricular arrhythmias without compromising safety. These exploratory findings support further evaluation of telemetry-guided outpatient heart failure management in larger, adequately powered multicenter randomized trials.

## Introduction

Heart failure (HF) remains a major cause of morbidity, mortality, and healthcare utilization worldwide despite substantial progress in pharmacological therapy. Contemporary guideline-directed medical therapy (GDMT), including angiotensin receptor-neprilysin inhibitors (ARNi), β-blockers (BB), mineralocorticoid receptor antagonists (MRA), and sodium-glucose cotransporter-2 (SGLT2) inhibitors, has consistently demonstrated reductions in mortality and HF hospitalization in patients with heart failure with reduced ejection fraction (HFrEF) [1–6]. These therapies exert complementary neurohormonal, hemodynamic, and myocardial protective effects and form the cornerstone of modern HF management.

Beyond progressive pump failure, arrhythmic events represent a major mode of death in HFrEF. Sudden cardiac death (SCD), most commonly mediated by ventricular tachycardia (VT) or ventricular fibrillation, accounts for approximately 30–50% of mortality in chronic HF populations despite state-of-the-art therapy [7]. Importantly, non-sustained and intermittent ventricular arrhythmias are frequently under-detected in standard outpatient follow-up, yet are strongly associated with increased risk of sustained VT, appropriate implantable cardioverter-defibrillator (ICD) therapies, and mortality [8].

Early initiation and rapid up-titration of GDMT have been shown to reduce both progressive HF and arrhythmic risk through reverse ventricular remodeling, autonomic stabilization, and improved myocardial electrophysiological properties [9–13]. However, real-world uptake remains poor, with prolonged delays in dose escalation and low achievement of guideline-recommended targets [14, 15]. Concerns regarding hypotension, bradycardia, renal dysfunction, electrolyte imbalance, and arrhythmic instability frequently limit aggressive optimization – particularly during early treatment phases [16].

Remote telemetric monitoring offers a strategy to address both safety and rhythm surveillance by enabling continuous assessment of hemodynamics and cardiac rhythm. The TIM-HF2 trial demonstrated improved clinical outcomes with structured remote HF management [17]; however, most telemonitoring programs have focused on congestion and symptom control, with limited emphasis on arrhythmia detection or rhythm-guided therapy escalation.

Given the central prognostic role of ventricular arrhythmias in HF, continuous rhythm surveillance during rapid GDMT up-titration may provide critical clinical benefit. Early identification of VT may enable timely primary or secondary prevention strategies, including ICD implantation, while real-time hemodynamic monitoring may support more assertive yet safe BB escalation – one of the most effective anti-arrhythmic interventions in HF [18].

The TELEMETRIC-HF study aimed to evaluate whether telemetric remote monitoring can facilitate accelerated GDMT up-titration while ensuring hemodynamic stability and improving arrhythmic surveillance. The study compared pharmacological dose escalation, arrhythmic event detection, clinical status, and biochemical safety between telemetry-guided management and standard care during early and intermediate follow-up. We hypothesized that continuous remote monitoring would permit faster BB optimization and enhanced detection of actionable arrhythmias.

## Methods

### Study Design and Setting

TELEMETRIC-HF is a single-center, randomized, open-label, parallel-group interventional trial conducted at the National Institute for Heart and Blood Vessels “Dedinje,” Belgrade, Serbia, a tertiary referral center specialized in advanced HF care. The study was designed to evaluate the efficacy and safety of rapid up-titration of GDMT in patients with HFrEF, guided either by telemetric remote monitoring or control group follow-up without telemetric monitoring. Patient enrollment was conducted between 1 May 2023 and 19 September 2025.

### Study Population

#### Inclusion Criteria

Eligible patients were adults aged 18–80 years with chronic HFrEF (left ventricular ejection fraction [LVEF] ≤40%) and New York Heart Association (NYHA) functional class II-III symptoms at screening. Patients were required to be receiving suboptimal GDMT, defined as ≤25% of the guideline-recommended target dose (TD) for ARNi or angiotensin-converting enzyme inhibitor/angiotensin receptor blocker and BB, and ≤50% TD for MRA.

#### Exclusion Criteria

Patients were excluded if any of the following criteria were present:

1. Age <18 or >80 years.
2. Documented intolerance to high-dose BB or renin-angiotensin system inhibitors (angiotensin-converting enzyme inhibitor or angiotensin receptor blocker).
3. Requirement for invasive mechanical ventilation within 24 h prior to screening.
4. Significant pulmonary disease contributing to dyspnea or primary right HF.
5. Recent cardiovascular events or procedures, including myocardial infarction, unstable angina, cardiac surgery, or cardiac resynchronization therapy implantation within 3 months, or percutaneous coronary intervention within 1 month.
6. Acute HF precipitated by a clearly correctable cause.
7. Active myocarditis, uncorrected thyroid disease, or known infiltrative or hypertrophic cardiomyopathy.
8. History of heart transplantation, ventricular assist device implantation, or listing for transplantation.
9. Untreated sustained ventricular arrhythmias with syncope.
10. Hemodynamically significant valvular disease or left ventricular outflow tract obstruction.
11. Active infection at the time of screening.
12. Stroke or transient ischemic attack within the preceding 3 months.
13. Life-threatening liver disease.
14. Severe renal dysfunction (eGFR: <30 mL/min/1.73 m^2^) or need for dialysis.
15. Severe psychiatric or neurological disease, cirrhosis, or active malignancy with limited life expectancy.
16. Participation in another interventional clinical trial within 30 days prior to screening.
17. Anticipated prolonged hospitalization or discharge to a long-term care facility.
18. Inability to comply with study procedures.
19. Pregnancy or lactation.

### Randomization and Interventions

Participant flow through the study is summarized in the CONSORT flow diagram (Fig. 1). Of the 44 patients assessed for eligibility, 30 met the inclusion criteria and were randomized in a 1:1 ratio either to the telemetry group (*n* = 15) or the control group (*n* = 15). Randomization was performed using a computer-generated random allocation sequence. Owing to the exploratory pilot design, simple randomization without blocking or stratification was used. The study was open-label; therefore, treatment allocation was not concealed after randomization. All randomized patients received the allocated intervention and completed the 8-week follow-up. No participants were lost to follow-up, discontinued the intervention, or were excluded from the final analysis. The completed CONSORT 2025 checklist accompanying this randomized pilot trial is available in the online supplementary Materials (for all online suppl. material, see https://doi.org/10.1159/crd/absag008).

**Fig. 1.**
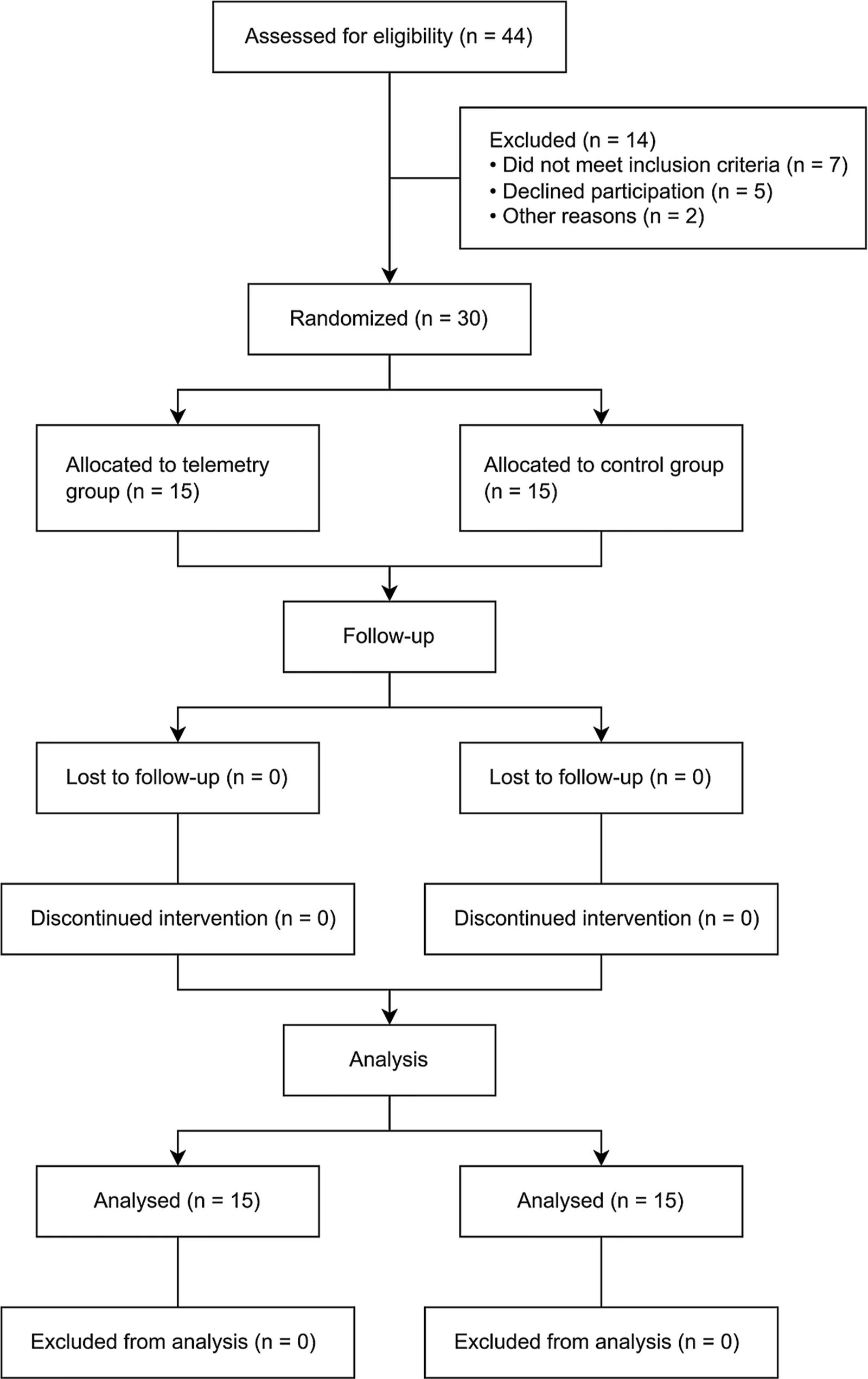
CONSORT flow diagram of participant enrollment, randomization, follow-up, and analysis in the TELEMETRIC-HF pilot study. A total of 44 patients were assessed for eligibility between May 2023 and September 2025. Fourteen patients were excluded, including 7 who did not meet the inclusion criteria, 5 who declined participation, and 2 for other reasons. Thirty eligible patients were randomized in a 1:1 ratio to telemetry (*n* = 15) or the control group (*n* = 15). All randomized participants received the allocated intervention, completed the 8-week follow-up, and were included in the final analysis, with no losses to follow-up, treatment discontinuations, or exclusions from analysis.

### GDMT Optimization Protocol

GDMT, including ARNi, BB, MRA, and SGLT2i, was optimized according to the 2021 ESC Guidelines for the Diagnosis and Treatment of Acute and Chronic Heart Failure [19] and the 2022 AHA/ACC/HFSA Guideline for the Management of Heart Failure [20]. All patients underwent rapid up-titration, aiming to achieve the guideline-recommended target or maximally tolerated doses within a 2-week period. At each scheduled assessment, eligibility for dose escalation was determined using a predefined protocol based on clinical status, blood pressure (BP), heart rate (HR), renal function, serum potassium concentration, and symptoms of medication intolerance. Dose escalation was withheld in the presence of symptomatic hypotension, systolic BP of <90 mm Hg, resting HR of <50 beats/min, clinically significant bradyarrhythmias or ventricular arrhythmias requiring further evaluation, serum potassium of >5.5 mmol/L, a ≥30% increase in serum creatinine from baseline, or worsening HF requiring intravenous therapy or hospitalization. The same titration algorithm was applied in both study groups; however, patients assigned to the telemetry group additionally underwent continuous remote monitoring, enabling earlier identification of clinically significant events and unscheduled clinical reassessment when required. Loop diuretic therapy was adjusted individually to maintain euvolemia, as current ESC guidelines do not recommend a TD for these agents.

### Telemetry Workflow

Patients assigned to the telemetry-guided group were instructed to wear the CPC12 ambulatory telemonitoring device (Checkpoint Cardio Ltd., Bulgaria) continuously throughout the 8-week study period. The system enabled continuous remote assessment of ECG rhythm, including HR and rhythm (sinus rhythm vs. atrial fibrillation [AF]), QRS duration and morphology, QTc interval, ventricular ectopy and VT, as well as BP, oxygen saturation, body temperature, and physical activity. Physiological data were transmitted to a secure cloud-based platform and reviewed by the study team. Automated alerts for clinically significant arrhythmias or abnormal physiological parameters triggered timely clinical assessment, with patient contact and intervention when clinically indicated. Potentially artefactual or non-actionable alerts were verified by reviewing the original telemetry recordings before any clinical action was undertaken. Medication adjustments and recommendations for hospital evaluation were made by experienced HF specialists at the National Institute for Heart and Blood Vessels “Dedinje” based on the overall clinical assessment. Patients assigned to the control group received routine outpatient follow-up without continuous telemetric monitoring, while GDMT optimization was performed according to the same predefined titration protocol in both groups.

### Definition of VT

VT was defined according to the 2022 ESC Guidelines for the management of patients with ventricular arrhythmias and the prevention of SCD [8] as three or more consecutive ventricular beats originating below the His bundle at a rate exceeding 100 beats/min. Episodes lasting <30 s were classified as non-sustained ventricular tachycardia, whereas episodes lasting ≥30 s or requiring earlier termination because of hemodynamic compromise were classified as sustained VT. Both symptomatic and asymptomatic ventricular arrhythmias detected by continuous telemetry were recorded. All recordings suggestive of VT were independently reviewed and confirmed by two experienced cardiologists with expertise in HF and cardiac electrophysiology prior to any clinical management decisions.

### Follow-Up and Assessments

Clinical assessment, including evaluation of HF signs and symptoms, was performed at baseline and at 1-, 4-, and 8-week follow-up visits. Laboratory testing, including routine biochemistry and biomarkers, was obtained at the same time points. GDMT doses were reviewed and adjusted at each visit when clinically and biochemically appropriate.

All patients were followed for 8 weeks after randomization. Final outcome assessment was performed at week 8 and included documentation of hospitalizations, medication changes, adverse events, and patient-reported outcomes. In the telemetry group, additional data on telemetry system tolerability and wearing comfort were collected.

### Endpoints of the Study

*The primary endpoint* was the between-group difference in BB optimization at 8 weeks, defined as the achieved percentage of the guideline-recommended TD of bisoprolol. A clinically relevant treatment effect was prespecified as an absolute between-group difference of ≥20% of target BB dose at 8 weeks.

*Secondary endpoints* included:

- GDMT optimization: achieved percentage of TD for ARNi, BB, MRA, and SGLT2 inhibitor at 1, 2, 4, and 8 weeks.
- Arrhythmia detection: incidence of telemetry-detected VT, AF, paroxysmal supraventricular tachycardia (PSVT), bradycardia <60/min, and tachycardia >100/min.
- Safety/tolerability: hypotension, bradycardia, renal dysfunction, electrolyte abnormalities, electrode allergy, telemetry technical failure, and wearing comfort.
- Clinical status: NYHA class change from baseline to 8 weeks.
- Quality of life (QoL): Minnesota Living with Heart Failure Questionnaire (MLHFQ) category/score change from baseline to 8 weeks.
- Biomarker response: NT-proBNP change from baseline to 8 weeks.
- Healthcare utilization: unscheduled calls, unscheduled visits, arrhythmia-related hospitalization, HF hospitalization, and ICD implantation.

### Sample Size Rationale

This study was designed as an exploratory pilot randomized trial to evaluate the feasibility, safety, and potential clinical utility of telemetry-guided optimization of GDMT in patients with HFrEF. No formal a priori sample size calculation was performed because the primary objective was to generate preliminary estimates of treatment effect and assess the feasibility of the intervention rather than to definitively evaluate clinical efficacy. A total sample size of 30 patients (15 per group) was considered pragmatic and appropriate for this investigator-initiated pilot study, providing sufficient data to evaluate study procedures, intervention adherence, safety, and variability of the primary endpoint while informing the design and sample size calculations of future adequately powered multicenter randomized trials. An absolute between-group difference of ≥20% in achievement of the guideline-recommended BB TD at 8 weeks was prespecified as a clinically meaningful treatment effect for interpretation of the primary endpoint.

### Statistical Analysis

Categorical variables are presented as frequencies and percentages and were compared using the χ^2^ test or Fisher’s exact test, as appropriate. Continuous variables are expressed as mean ± standard deviation or median with interquartile range, depending on data distribution, which was assessed using the Shapiro-Wilk test. Between-group comparisons were performed using the independent-samples Student’s *t* test or the Mann-Whitney U test, as appropriate. For the primary endpoint, the absolute between-group difference and its corresponding 95% confidence interval (CI) were estimated using univariable linear regression with treatment group as the independent variable. Owing to the exploratory nature and limited sample size of this pilot study, longitudinal mixed-effects or repeated-measures analyses were not performed, as such models were unlikely to provide robust parameter estimates. Secondary endpoint analyses were considered exploratory, and no adjustment for multiple comparisons was applied. All statistical tests were two-sided, and a *p* value of <0.05 was considered statistically significant. Statistical analyses were performed using IBM SPSS Statistics, version 31.0 (IBM Corp., Armonk, NY, USA).

## Results

A total of 30 patients (22 [73.3%] males; median age 60 [44, 69] years) with chronic HF were included, with 15 (11 [73.3%] males; mean age 54.5 ± 15.8 years) assigned to the telemetry group and 15 (11 [73.3%] males; mean age 56.0 ± 16.0 years) to the control group. The two groups were well matched with respect to demographic variables, comorbidities, HF severity, background therapy, echocardiographic parameters, and laboratory findings (Table 1).

**Table 1.** Baseline characteristics

Parameters	Telemetry group (*n* = 15)	Control group (*n* = 15)	*p* value
*Demographics*			
Age at study enrollment, mean±SD	54.5±15.8	56.0±16.0	0.794
Age at HF diagnosis, mean±SD	50.5±14.7	52.7±15.0	0.682
Sex, *n* (%)	​	​	1.000
Male	11 (73.3)	11 (73.3)	
Female	4 (26.7)	4 (26.7)	
BMI, mean±SD	26.8±4.9	25.7±6.2	0.576
*Clinical characteristics*			
Heart failure etiology, *n* (%)	​	​	0.139
ICM	4 (26.7)	9 (60.0)	
DCM	11 (73.3)	6 (40.0)	
NYHA class, *n* (%)	​	​	1.000
I	0 (0)	0 (0)	
II	12 (80)	13 (86.7)	
III	3 (20)	2 (13.3)	
DM, *n* (%)	3 (20)	3 (20)	1.000
HTA, *n* (%)	9 (60)	8 (53.3)	χ^2^ = 0.136
			0.713
Peripheral vascular disease, *n* (%)	0 (0)	0 (0)	NA
COPD, *n* (%)	1 (6.7)	1 (6.7)	1.000
COPD treated with bronchodilator/prednisone, *n* (%)	1 (6.7)	1 (6.7)	1.000
Smoker (current/former), *n* (%)	3 (20)/4 (26.7)	1 (6.7)/6 (40)	χ^2^ = 1.4
			0.497
Previous MI, *n* (%)	4 (26.7)	3 (20)	1.000
Previous stroke, *n* (%)	2 (13.3)	1 (6.7)	1.000
eGFR <60 mL/min/1.73 m^2^, *n* (%)	5 (33.3)	3 (20)	0.682
MLHFQ score, *n* (%)	​	​	1.000
<24 = good QoL	0 (0)	0 (0)	
25–45 = moderate impairment	12 (80)	13 (86.7)	
>45 = severe impairment	3 (20)	2 (13.3)	
Systolic blood pressure, mm Hg, mean±SD	118±14	119±13	0.969
Diastolic blood pressure, mm Hg, mean±SD	74±9	72±8	0.441
HR (bpm), mean±SD	75±14	68±10	0.111
*Hospitalizations*			
Hospitalized in the last 12 months, *n* (%)	6 (40)	2 (13.3)	0.215
≥2 hospitalizations lifetime, *n* (%)	2 (13.3)	1 (6.7)	1.000
*HF therapy before inclusion*			
ARNi, *n* (%)	15 (100)	15 (100)	NA
Sacubitril/valsartan dose (mg), mean±SD	100±0	100±0	NA
BB, *n* (%)	15 (100)	15 (100)	NA
Bisoprolol dose (mg), mean±SD	2.0±0.6	2.3±0.5	0.247
MRA, *n* (%)	14 (93.3)	14 (93.3)	1.000
Spironolactone/eplerenone dose (mg), mean±SD	25±0	25±0	NA
SGLT2 inhibitor, *n* (%)	13 (86.7)	14 (93.3)	1.000
Dapagliflozin/empagliflozin dose (mg), mean±SD	10±0	10±0	NA
Diuretic, *n* (%)	15 (100)	14 (93.3)	1.000
*TTE*			
LVEDD, mm, mean±SD	67.3±9.2	65.1±7.8	0.500
LVEDD index, mm/m^2^, mean±SD	33.4±4.1	32.9±5.6	0.775
LVESD, mm, mean±SD	53.2±8.9	51.9±7.5	0.677
LVESD index, mm/m^2^, mean±SD	26.4±3.8	26.3±5.4	0.960
LVEDV, mL, Me [IQR]	174 [108; 240]	187 [136; 237]	0.838
LVEDVi, mL/m^2^, mean±SD	105.1±52.7	99.3±32.3	0.719
LVESV, mL, Me [IQR]	113 [62; 165]	138 [94; 182]	0.902
LVESVi, mL/m^2^, Me [IQR]	60 [31.5; 88.5]	68.5 [47.9; 89.1]	0.870
LVEF, %, mean±SD	26.5±8.0	30.9±5.7	0.094
GLS, %, Me [IQR]	−12.3 [-9.8; −14.9]	−11.3 [-7.5; −15.1]	0.870
FAC, %, mean±SD	35.4±9.3	35.1±6.9	0.912
TAPSE, mm, mean±SD	19.1±4.0	19.5±4.3	0.795
*Laboratory parameters*			
Hemoglobin, g/L, mean±SD	145.2±17.6	142.1±13.4	0.587
Erythrocytes (Er), ×10^12^/L, mean±SD	5.0±0.5	4.9±0.4	0.507
Platelets, ×10^9^/L, mean±SD	233.5±49.1	226.0±54.2	0.695
Sodium, mmol/L, mean±SD	139.5±2.2	139.6±1.7	0.852
Potassium, mmol/L, Me [IQR]	4.5 [4.2; 4.8]	4.5 [4.3; 4.7]	0.595
Calcium, mmol/L, mean±SD	2.39±0.092	2.40±0.091	0.739
Magnesium, mmol/L, mean±SD	0.9±0.05	0.8±0.2	0.614
Chloride, mmol/L, mean±SD	103.9±2.6	102.3±2.6	0.101
Phosphate, mmol/L, mean±SD	1.1±0.2	1.2±0.2	0.200
Bicarbonate, mmol/L, mean±SD	28.1±3.3	28.3±2.0	0.876
Total bilirubin, µmol/L, Me [IQR]	11.1 [7.6; 14.7]	10.4 [6.7; 14.2]	0.838
Urea, mmol/L, Me [IQR]	7.3 [5.4; 9.3]	6.6 [5.7; 7.5]	0.567
Creatinine, µmol/L, Me [IQR]	89.4 [66.3; 112.4]	75.9 [53.3; 98.5]	0.098
Glucose, mmol/L, Me [IQR]	5.9 [5.0; 6.9]	5.4 [4.9; 6.0]	0.775
ALT, U/L, mean±SD	26.0±12.5	25.6±9.8	0.923
AST, U/L, mean±SD	24.5±6.0	26.7±8.5	0.433
LDH, U/L, mean±SD	382.0±68.7	364.7±49.2	0.435
CK, U/L, Me [IQR]	81 [60; 101]	101 [50; 151]	0.187
Total protein, g/L, mean±SD	71.0±3.3	72.7±4.6	0.240
Albumin, g/L, mean±SD	43.7±3.0	44.0±3.5	0.824
NT-proBNP, pg/mL, Me [IQR]	595 [279; 1,339]	671 [222; 912]	0.683

ALT, alanine aminotransferase; ARNi, angiotensin receptor-neprilysin inhibitor; AST, aspartate aminotransferase; BB, beta-blocker; BMI, body mass index; bpm, beats per minute; CK, creatine kinase; COPD, chronic obstructive pulmonary disease; DCM, dilated cardiomyopathy; DM, diabetes mellitus; eGFR, estimated glomerular filtration rate; Er, erythrocytes; FAC, fractional area change; GLS, global longitudinal strain; HF, heart failure; HTA, arterial hypertension; ICM, ischemic cardiomyopathy; IQR, interquartile range; LDH, lactate dehydrogenase; LVEDD, left ventricular end-diastolic diameter; LVEDV, left ventricular end-diastolic volume; LVEDVi, left ventricular end-diastolic volume index; LVEF, left ventricular ejection fraction; LVESD, left ventricular end-systolic diameter; LVESV, left ventricular end-systolic volume; LVESVi, left ventricular end-systolic volume index; MI, myocardial infarction; MLHFQ, Minnesota Living with Heart Failure Questionnaire; MRA, mineralocorticoid receptor antagonist; NA, not applicable; NT-proBNP, N-terminal pro-B-type natriuretic peptide; NYHA, New York Heart Association; QoL, quality of life; SD, standard deviation; SGLT2, sodium–glucose cotransporter 2; TAPSE, tricuspid annular plane systolic excursion; TTE, transthoracic echocardiography.

Most patients were in NYHA class II (80.0% vs. 86.7%), while 20.0% and 13.3%, respectively, were in NYHA class III (*p* = 1.000) (Fig. 2). Ischemic cardiomyopathy was numerically more frequent in controls (60.0% vs. 26.7%), whereas dilated cardiomyopathy predominated in the telemetry group (73.3% vs. 40.0%; *p* = 0.139). Comorbidities, MLHFQ categories (Fig. 3), BP, and resting HR were comparable. Hospitalization during the preceding 12 months was numerically more frequent in the telemetry group (40.0% vs. 13.3%; *p* = 0.215). All patients were receiving ARNI and BB therapy before inclusion. MRA and SGLT2i use was also comparable. Baseline sacubitril/valsartan doses were identical, while mean bisoprolol doses were 2.0 ± 0.6 and 2.3 ± 0.5 mg, respectively (*p* = 0.247).

**Fig. 2.**
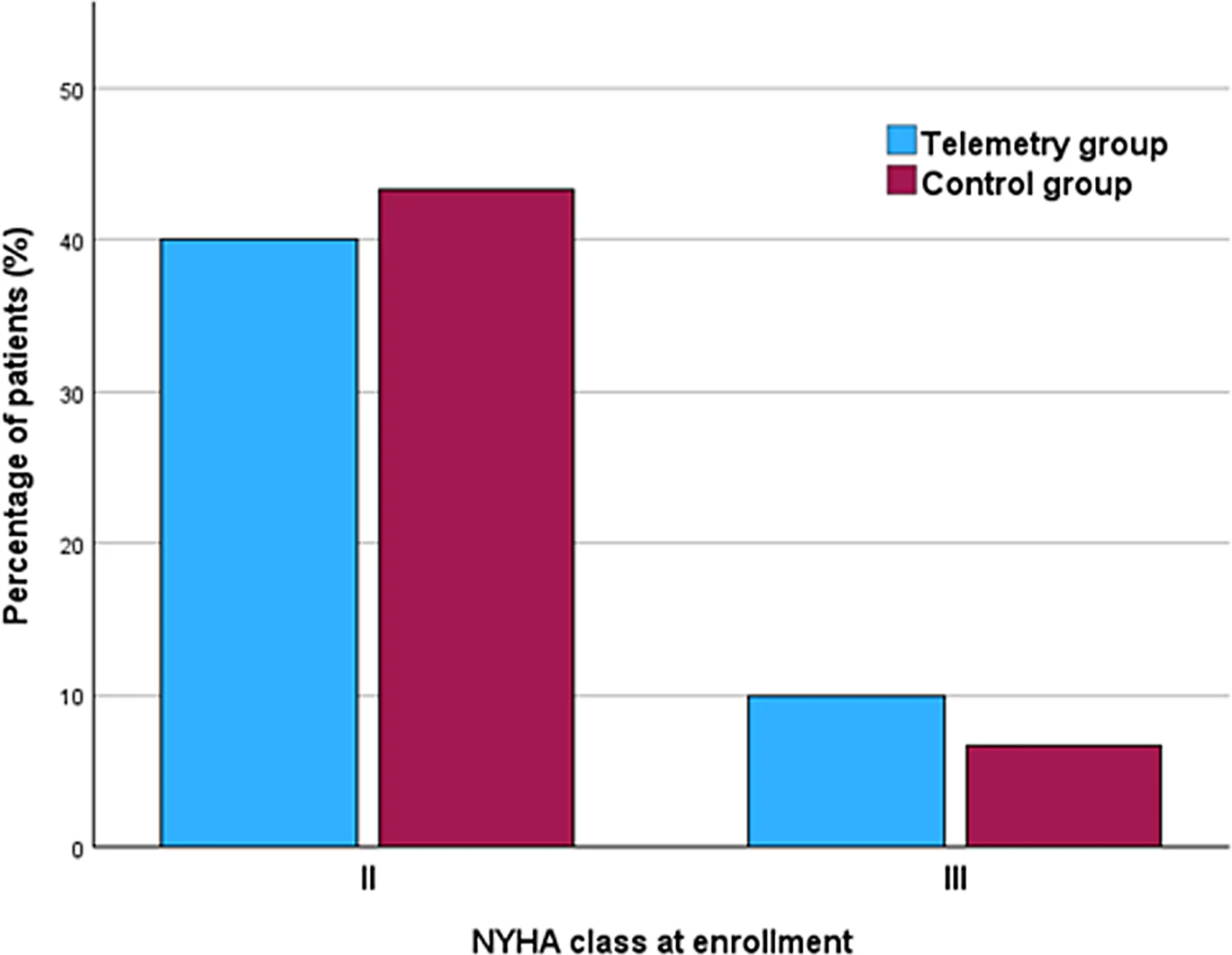
Distribution of NYHA functional class at baseline according to study group. The distribution of NYHA functional class was comparable between the telemetry and control groups at enrollment (*p* = 1.000).

**Fig. 3.**
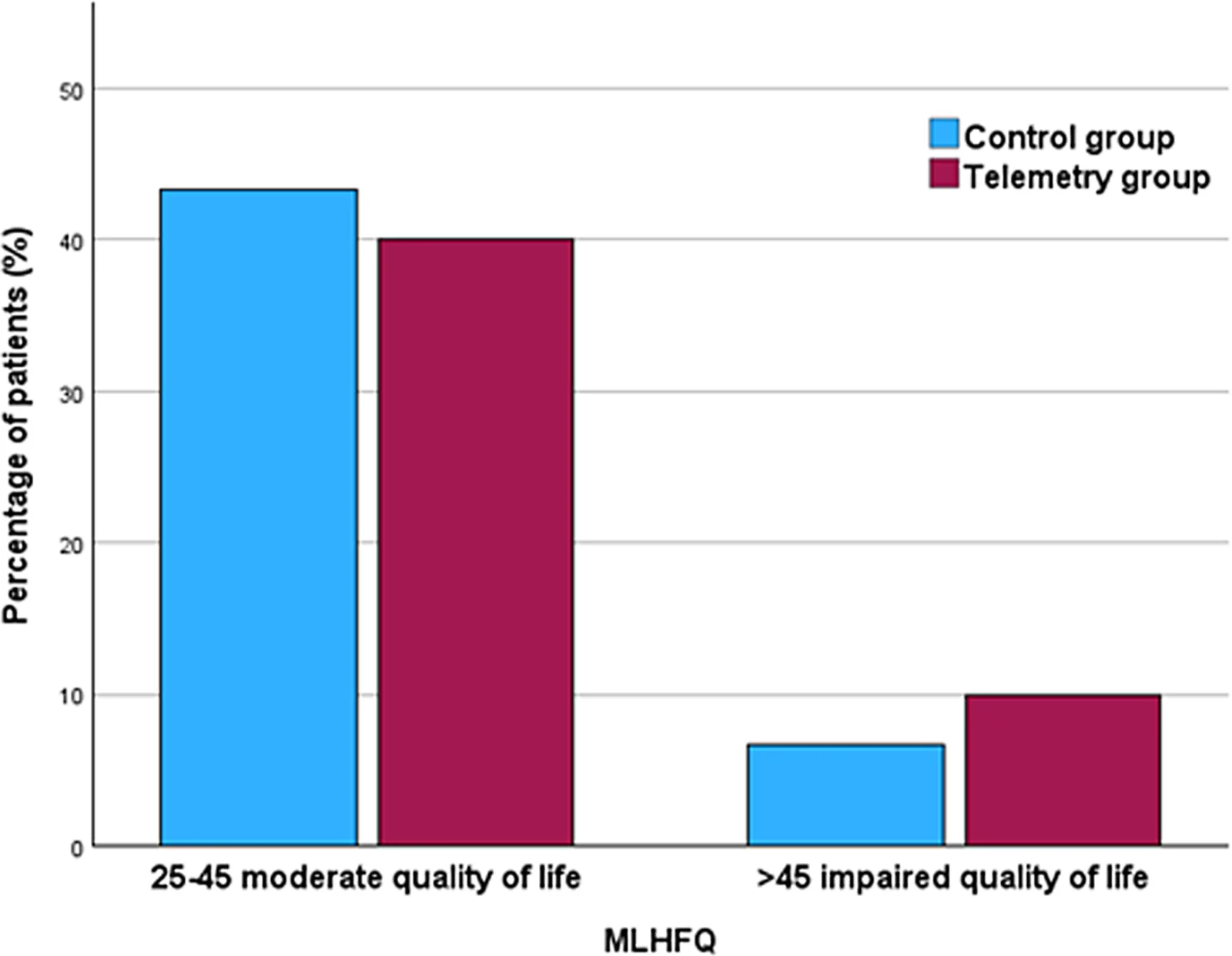
Distribution of MLHFQ categories at baseline according to study group. The proportions of patients with moderate (25–45 points) and impaired (>45 points) health-related QoL were comparable between the telemetry and control groups at enrollment (*p* = 1.000).

Both groups had severe LV systolic dysfunction. Mean LVEF was numerically lower in the telemetry group (26.5 ± 8.0% vs. 30.9 ± 5.7%; *p* = 0.094), whereas other echocardiographic parameters were comparable. No significant differences were observed in baseline laboratory parameters, including NT-proBNP concentrations (595 [279, 1,339] vs. 671 [222, 912] pg/mL; *p* = 0.683) (Fig. 4).

**Fig. 4.**
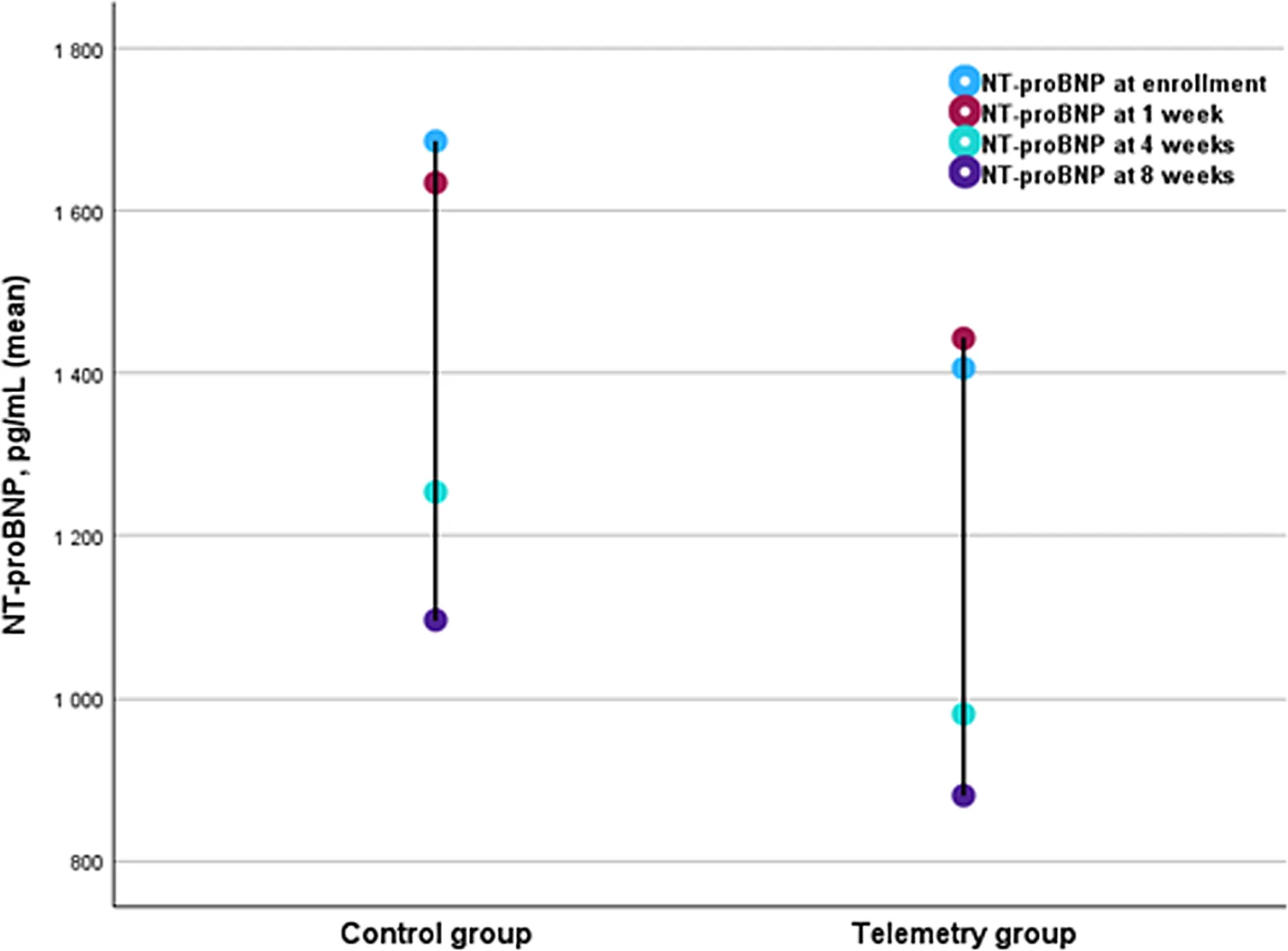
Temporal changes in NT-proBNP levels in the telemetry and control groups. NT-proBNP concentrations at enrollment and at 1, 4, and 8 weeks of follow-up in the telemetry and control groups. A progressive decline over time is observed in both groups, with numerically lower levels at 8 weeks in the telemetry group.

During the 1-week follow-up, clinical status remained comparable between groups (Table 2). Most patients were classified as NYHA II (80.0% vs. 86.7%), with no significant differences in NYHA class distribution, SBP, DBP, or resting HR. Most laboratory parameters also remained similar; however, serum sodium (140.7 ± 3.2 vs. 138.5 ± 1.4 mmol/L, *p* = 0.024) and chloride (104.5 ± 2.4 vs. 102.4 ± 2.2 mmol/L, *p* = 0.016) were modestly higher in the telemetry group. Renal function, liver enzymes, metabolic parameters, and NT-proBNP did not differ significantly between groups.

**Table 2.** Follow-up characteristics by study group during the 1-week telemetry monitoring period

Parameters	Telemetry group (*n* = 15)	Control group (*n* = 15)	*p* value
*Clinical characteristics*			
NYHA class, *n* (%)	​	​	1.000
I	0 (0)	0 (0)	
II	12 (80)	13 (86.7)	
III	3 (20)	2 (13.3)	
Systolic blood pressure, mm Hg, mean±SD	117±11	113±10	0.316
Diastolic blood pressure, mm Hg, mean±SD	71±8	71±7	0.904
HR (bpm), mean±SD	74±11	69±8	0.183
*Laboratory parameters*			
Hemoglobin, g/L, mean±SD	144.5±16.7	142.6±13.7	0.731
Erythrocytes (Er), ×10^12^/L, mean±SD	4.9±0.5	4.8±0.4	0.603
Platelets, ×10^9^/L, mean±SD	250.8±54.1	218.8±50.3	0.105
Sodium, mmol/L, mean±SD	140.7±3.2	138.5±1.4	0.024
Potassium, mmol/L, Me [IQR]	4.6 [4.3; 5.0]	4.5 [4.2; 4.9]	0.367
Calcium, mmol/L, mean±SD	2.44±0.2	2.38±0.1	0.293
Magnesium, mmol/L, mean±SD	0.9±0.06	0.9±0.1	0.650
Chloride, mmol/L, mean±SD	104.5±2.4	102.4±2.2	0.016
Phosphate, mmol/L, mean±SD	1.1±0.1	1.2±0.2	0.343
Bicarbonate, mmol/L, mean±SD	27.8±2.6	27.6±2.2	0.798
Total bilirubin, µmol/L, Me [IQR]	8.8 [7.9; 13.7]	13.2 [10.0; 14.5]	0.161
Urea, mmol/L, Me [IQR]	8.4 [6.3; 9.2]	6.6 [5.8; 7.9]	0.217
Creatinine, µmol/L, Me [IQR]	87.8 [76.3; 115.1]	75.3 [72.9; 87.5]	0.202
Glucose, mmol/L, Me [IQR]	6.1 [5.6; 6.5]	5.7 [5.4; 6.3]	0.345
ALT, U/L, mean±SD	25.7±9.9	27.4±12.6	0.691
AST, U/L, mean±SD	23.9±4.1	28.1±10.6	0.157
LDH, U/L, mean±SD	380.5±38.4	362.2±40.7	0.215
CK, U/L, Me [IQR]	89 [80; 104]	124 [89; 183]	0.148
Total protein, g/L, mean±SD	72.8±3.9	72,5±3,5	0.848
Albumin, g/L, mean±SD	45.4±4.1	44.3±3.2	0.441
NT-proBNP, pg/mL, Me [IQR]	504.0 [244.0; 1,113.0]	367 [146.5; 1,201.5]	0.870
*Arrhythmia surveillance*			
AF, *n* (%)	0 (0)	0 (0)	NA
VT, *n* (%)	2 (13.3)	0 (0)	0.483
PSVT, *n* (%)	0 (0)	0 (0)	NA
Bradycardia <60/min, *n* (%)	0 (0)	0 (0)	NA
Tachycardia >100/min, *n* (%)	0 (0)	0 (0)	NA
*Telemetry safety and adherence*			
Telemetry technical issue, *n* (%)	0 (0)	–	NA
Allergy to electrodes, *n* (%)	0 (0)	–	NA
Wearing comfort score, *n* (%)	​	–	NA
1 – unacceptable	1 (6.7)		
5 – maximally acceptable	14 (93.3)		
*Clinical events*			
Hospitalization due to HF, *n* (%)	0 (0)	0 (0)	NA
Hospitalization due to arrhythmia, *n* (%)	1 (6.7)	0 (0)	1.000
Unscheduled calls, *n* (%)	2 (13.3)	0 (0)	0.483
Reason for unscheduled call, *n* (%)	​	​	0.483
Hypotension	0 (0)	0 (0)	
Arrhythmia	2 (13.3)	0 (0)	
Edema	0 (0)	0 (0)	
Dyspnea	0 (0)	0 (0)	
Medication adjustment required, *n* (%)	1 (6.7)	0 (0)	1.000
Unscheduled visits, *n* (%)	1 (6.7)	0 (0)	1.000

AF, atrial fibrillation; ALT, alanine aminotransferase; AST, aspartate aminotransferase; bpm, beats per minute; CK, creatine kinase; Er, erythrocytes; HF, heart failure; IQR, interquartile range; LDH, lactate dehydrogenase; NA, not applicable; NT-proBNP, N-terminal pro-B-type natriuretic peptide; NYHA, New York Heart Association; PSVT, paroxysmal supraventricular tachycardia; SD, standard deviation; VT, ventricular tachycardia.

VT was detected in 2 patients (13.3%) in the telemetry group, whereas no ventricular arrhythmias were identified in the control group (*p* = 0.483). No episodes of AF, PSVT, clinically significant bradycardia, or tachycardia occurred in either group. Telemetry was well tolerated, with no technical failures or allergic reactions, and 93.3% of patients rated device comfort as maximal. One arrhythmia-related hospitalization occurred in the telemetry group, accompanied by two unscheduled telephone consultations, one unscheduled outpatient visit, and initiation of amiodarone therapy in 1 patient. No HF-related hospitalizations occurred in either group.

During the 1- to 4-week follow-up, clinical status remained comparable between groups (Table 3). NYHA functional class and MLHFQ distributions did not differ significantly, with most patients classified as NYHA II and reporting moderate impairment in QoL. SBP (120 ± 9 vs. 108 ± 13 mm Hg, *p* = 0.009) and resting HR (71 ± 7 vs. 65 ± 6 bpm, *p* = 0.035) were higher in the telemetry group, whereas DBP was similar between groups. Laboratory parameters, including NT-proBNP, remained stable without significant between-group differences.

**Table 3.** Follow-up characteristics by study group during the 1- to 4-week telemetry monitoring period

Parameters	Telemetry group (*n* = 15)	Control group (*n* = 15)	*p* value
*Clinical characteristics*			
NYHA class, *n* (%)	​	​	χ^2^ = 1.040
I	2 (13.3)	2 (13.3)	0.595
II	13 (86.7)	12 (80)	
III	0 (0)	1 (6.7)	
Systolic blood pressure, mm Hg	120±9	108±13	0.009
Diastolic blood pressure, mm Hg	71±7	66±9	0.086
HR (bpm)	71±7	65±6	0.035
MLHFQ score, *n* (%)	​	​	χ^2^ = 1.048
<24 = good QoL	4 (26.7)	2 (13.3)	0.592
25–45 = moderate impairment	10 (66.4)	11 (73.3)	
>45 = severe impairment	1 (6.7)	2 (13.3)	
*Laboratory parameters*			
Hemoglobin, g/L, mean±SD	143.4±17.8	142.1±13.7	0.828
Erythrocytes (Er), ×10^12^/L, mean±SD	4.9±0.5	4.8±0.5	0.742
Platelets, ×10^9^/L, mean±SD	238.7±56.4	215.4±41.1	0.206
Sodium, mmol/L, mean±SD	139.0±1.7	138.7±1.9	0.617
Potassium, mmol/L, Me [IQR]	4.6 [4.3; 4.8]	4.5 [4.3; 4.6]	0.174
Calcium, mmol/L, mean±SD	2.4±0.1	2.4±0.1	1.000
Magnesium, mmol/L, mean±SD	0.86±0.07	0.89±0.11	0.456
Chloride, mmol/L, mean±SD	103.4±2.8	103.0±2.6	0.692
Phosphate, mmol/L, mean±SD	1.1±0.3	1.2±0.2	0.687
Bicarbonate, mmol/L, mean±SD	27.9±2.6	27.5±2.2	0.692
Total bilirubin, µmol/L, Me [IQR]	9.1 [7.7; 13.9]	11.9 [9.0; 14.9]	0.267
Urea, mmol/L, Me [IQR]	7.8 [6.5; 10.1]	7.3 [5.8; 8.7]	0.305
Creatinine, µmol/L, Me [IQR]	97.5 [76.3; 126.3]	82.4 [66.5; 100.4]	0.389
Glucose, mmol/L, Me [IQR]	6.0 [5.0; 7.0]	5.6 [5.0; 6.2]	1.000
ALT, U/L, mean±SD	24.4±7.0	26.1±12.7	0.660
AST, U/L, mean±SD	24.6±7.2	27.8±9.5	0.309
LDH, U/L, mean±SD	372.6±63.6	381.7±50.5	0.667
CK, U/L, Me [IQR]	112 [90; 148]	118 [78; 196]	0.486
Total protein, g/L, mean±SD	72.8±5.2	72.8±3.9	0.971
Albumin, g/L, mean±SD	44.2±2.5	43.4±13.3	0.843
NT-proBNP, pg/mL, Me [IQR]	414 [204; 829]	261 [228; 1,119]	0.806
*TTE*			
LVEDD, mm, mean±SD	63.3±8.5	61.9±6.5	0.616
LVEDD index, mm/m^2^, mean±SD	31.5±4.0	31.3±5.4	0.912
LVESD, mm, mean±SD	51.5±10.0	50.7±7.7	0.808
LVESD index, mm/m^2^, mean±SD	25.6±4.5	25.7±5.7	0.941
LVEDV, mL, Me [IQR]	178 [112; 195]	180 [136; 204]	0.775
LVEDVi, mL/m^2^, mean±SD	97.3±57.1	88.1±29.5	0.584
LVESV, mL, Me [IQR]	117 [69; 140]	130 [92; 154]	0.512
LVESVi, mL/m^2^, Me [IQR]	63.8 [36.7; 73.7]	71.1 [40.1; 80.3]	0.486
LVEF, %, mean±SD	33.9±8.1	32.9±6.5	0.694
GLS, %, Me [IQR]	−12.7 [-10.0; −15.3]	−9.8 [-8.4; −11.3]	0.174
FAC, %, mean±SD	38.0±8.6	36.8±9.1	0.714
TAPSE, mm, mean±SD	20.1±2.5	19.7±9.1	0.745
*Arrhythmia surveillance*			
AF, *n* (%)	0 (0)	0 (0)	NA
VT, *n* (%)	3 (20)	0 (0)	0.224
PSVT, *n* (%)	0 (0)	0 (0)	NA
Bradycardia <60/min, *n* (%)	0 (0)	0 (0)	NA
Tachycardia >100/min, *n* (%)	0 (0)	0 (0)	NA
*Telemetry safety and adherence*			
Telemetry technical issue, *n* (%)	0 (0)	–	NA
Allergy to electrodes, *n* (%)	1 (6.7)	–	NA
Wearing comfort score, *n* (%)	​	–	NA
1 – unacceptable	1 (6.7)		
5 – maximally acceptable	14 (93.3)		
*Clinical events*			
Hospitalization due to HF, *n* (%)	0 (0)	0 (0)	NA
Hospitalization due to arrhythmia, *n* (%)	2 (13.3)	0 (0)	0.483
Unscheduled calls, *n* (%)	3 (20)	0 (0)	0.224
Reason for unscheduled call, *n* (%)	​	​	0.224
Hypotension	0 (0)	0 (0)	
Arrhythmia	3 (20)	0 (0)	
Edema	0 (0)	0 (0)	
Dyspnea	0 (0)	0 (0)	
Medication adjustment required, *n* (%)	2 (13.3)	0 (0)	0.483
Unscheduled visits, *n* (%)	2 (13.3)	0 (0)	0.483

AF, atrial fibrillation; ALT, alanine aminotransferase; AST, aspartate aminotransferase; bpm, beats per minute; CK, creatine kinase; Er, erythrocytes; FAC, fractional area change; GLS, global longitudinal strain; HF, heart failure; IQR, interquartile range; LDH, lactate dehydrogenase; LVEDD, left ventricular end-diastolic diameter; LVEDV, left ventricular end-diastolic volume; LVEDVi, left ventricular end-diastolic volume index; LVEF, left ventricular ejection fraction; LVESD, left ventricular end-systolic diameter; LVESV, left ventricular end-systolic volume; LVESVi, left ventricular end-systolic volume index; MLHFQ, Minnesota Living with Heart Failure Questionnaire; NA, not applicable; NT-proBNP, N-terminal pro-B-type natriuretic peptide; NYHA, New York Heart Association; PSVT, paroxysmal supraventricular tachycardia; SD, standard deviation; TAPSE, tricuspid annular plane systolic excursion; TTE, transthoracic echocardiography; VT, ventricular tachycardia.

Echocardiographic parameters, including LV size, systolic function, GLS, and RV function, were comparable between groups, with no significant changes during follow-up. VT was detected in three telemetry patients (20.0%), while no ventricular arrhythmias occurred in the control group (*p* = 0.224).

No episodes of AF, PSVT, clinically significant bradycardia, or tachycardia were observed in either cohort. Telemetry was well tolerated, with only one mild electrode-related allergic reaction and high patient-reported comfort. Two arrhythmia-related hospitalizations, three unscheduled telephone consultations, and two unscheduled outpatient visits occurred in the telemetry group, with amiodarone initiated in 2 patients. No HF-related hospitalizations occurred in either group.

During the 4- to 8-week follow-up, functional status showed a nonsignificant trend toward improvement in the telemetry group (Table 4), with more patients achieving NYHA I (60.0% vs. 26.7%) and only one control patient remaining in NYHA III (*p* = 0.141; Fig. 5). MLHFQ category distribution was also comparable, although good QoL was more frequent in the telemetry group (46.7% vs. 25.7%, *p* = 0.496; Fig. 6). Hemodynamic parameters remained stable, with similar SBP and DBP, while resting HR was higher in the telemetry group (72 ± 9 vs. 64 ± 6 bpm, *p* = 0.008). Most laboratory parameters remained comparable. Platelet count (244.1 ± 48.7 vs. 195.5 ± 75.8 ×10^9^/L, *p* = 0.046), serum chloride (104.1 ± 3.0 vs. 101.9 ± 2.2 mmol/L, *p* = 0.032), and creatinine (95.5 [79.2, 128.0] vs. 74.1 [64.1, 93.1] µmol/L, *p* = 0.041) were higher in the telemetry group, whereas NT-proBNP and other laboratory parameters did not differ significantly. During this period, 1 telemetry patient (6.7%) required an unscheduled telephone consultation, an outpatient visit, arrhythmia-related hospitalization, and primary prevention ICD implantation, while no such events occurred in the control group.

**Table 4.** Follow-up characteristics by study group during the 4–8-week telemetry monitoring period

Parameters	Telemetry group (*n* = 15)	Control group (*n* = 15)	*p* value
*Clinical characteristics*			
NYHA class, *n* (%)	​	​	χ^2^ = 3.923
I	9 (60)	4 (26.7)	0.141
II	6 (40)	10 (66.7)	
III	0 (0)	1 (6.6)	
Systolic blood pressure, mm Hg, mean±SD	120±16	111±9	0.085
Diastolic blood pressure, mm Hg, mean±SD	71±7	67±7	0.128
HR (bpm), mean±SD	72±9	64±6	0.008
MLHFQ score, *n* (%)	​	​	χ^2^ = 1.402
<24 = good QoL	7 (46.7)	4 (26.7)	0.496
25–45 = moderate impairment	7 (46.7)	9 (60.0)	
>45 = severe impairment	1 (6.6)	2 (13.3)	
*Laboratory parameters*			
Hemoglobin, g/L, mean±SD	142.3±15.8	143.7±15.1	0.815
Erythrocytes (Er), ×10^12^/L, mean±SD	4.85±0.46	4.87±0.51	0.904
Platelets, ×10^9^/L, mean±SD	244.1±48.7	195.5±75.8	0.046
Sodium, mmol/L, mean±SD	139.9±4.0	136.3±9.0	0.170
Potassium, mmol/L, Me [IQR]	4.6 [4.4; 4.8]	4.5 [4.4; 4.7]	0.683
Calcium, mmol/L, mean±SD	2.4±0.11	2.4±0.07	0.854
Magnesium, mmol/L, mean±SD	0.9±0.06	0.9±0.12	0.661
Chloride, mmol/L, mean±SD	104.1±3.0	101.9±2.2	0.032
Phosphate, mmol/L, mean±SD	1.1±0.2	1.2±0.2	0.353
Bicarbonate, mmol/L, mean±SD	28.0±2.7	27.9±3.2	0.920
Total bilirubin, µmol/L, Me [IQR]	9.0 [8.3; 12.7]	13.0 [9.3; 16.1]	0.217
Urea, mmol/L, Me [IQR]	7.8 [6.2; 8.6]	7.3 [5.5; 8.9]	0.595
Creatinine, µmol/L, Me [IQR]	95.5 [79.2; 128.0]	74.1 [64.1; 93.1]	0.041
Glucose, mmol/L, Me [IQR]	5.8 [5.7; 6.7]	6.2 [5.3; 8.3]	0.935
ALT, U/L, mean±SD	27.5±8.2	25.2±9.4	0.475
AST, U/L, mean±SD	25.9±6.2	26.8±8.4	0.732
LDH, U/L, mean±SD	362.0±46.3	376.9±53.4	0.422
CK, U/L, Me [IQR]	115 [90; 144]	92 [69; 160]	0.539
Total protein, g/L, mean±SD	72.7±4.5	71.9±9.6	0.469
Albumin, g/L, mean±SD	44.5±2.5	44.3±2.8	0.830
NT-proBNP, pg/mL, Me [IQR]	334 [221; 756]	318 [202; 789]	0.902
*Clinical events*			
Unscheduled calls, *n* (%)	1 (6.7)	0 (0)	1.000
Number of unscheduled visits, *n* (%)	1 (6.7)	0 (0)	1.000
Hospitalization due to arrhythmia, *n* (%)	1 (6.7)	0 (0)	1.000
ICD implantation, *n* (%)	1 (6.7)	0 (0)	1.000

ALT, alanine aminotransferase; AST, aspartate aminotransferase; bpm, beats per minute; CK, creatine kinase; Er, erythrocytes; HF, heart failure; ICD, implantable cardioverter-defibrillator; IQR, interquartile range; LDH, lactate dehydrogenase; MLHFQ, Minnesota Living with Heart Failure Questionnaire; NA, not applicable; NT-proBNP, N-terminal pro-B-type natriuretic peptide; NYHA, New York Heart Association; SD, standard deviation.

**Fig. 5.**
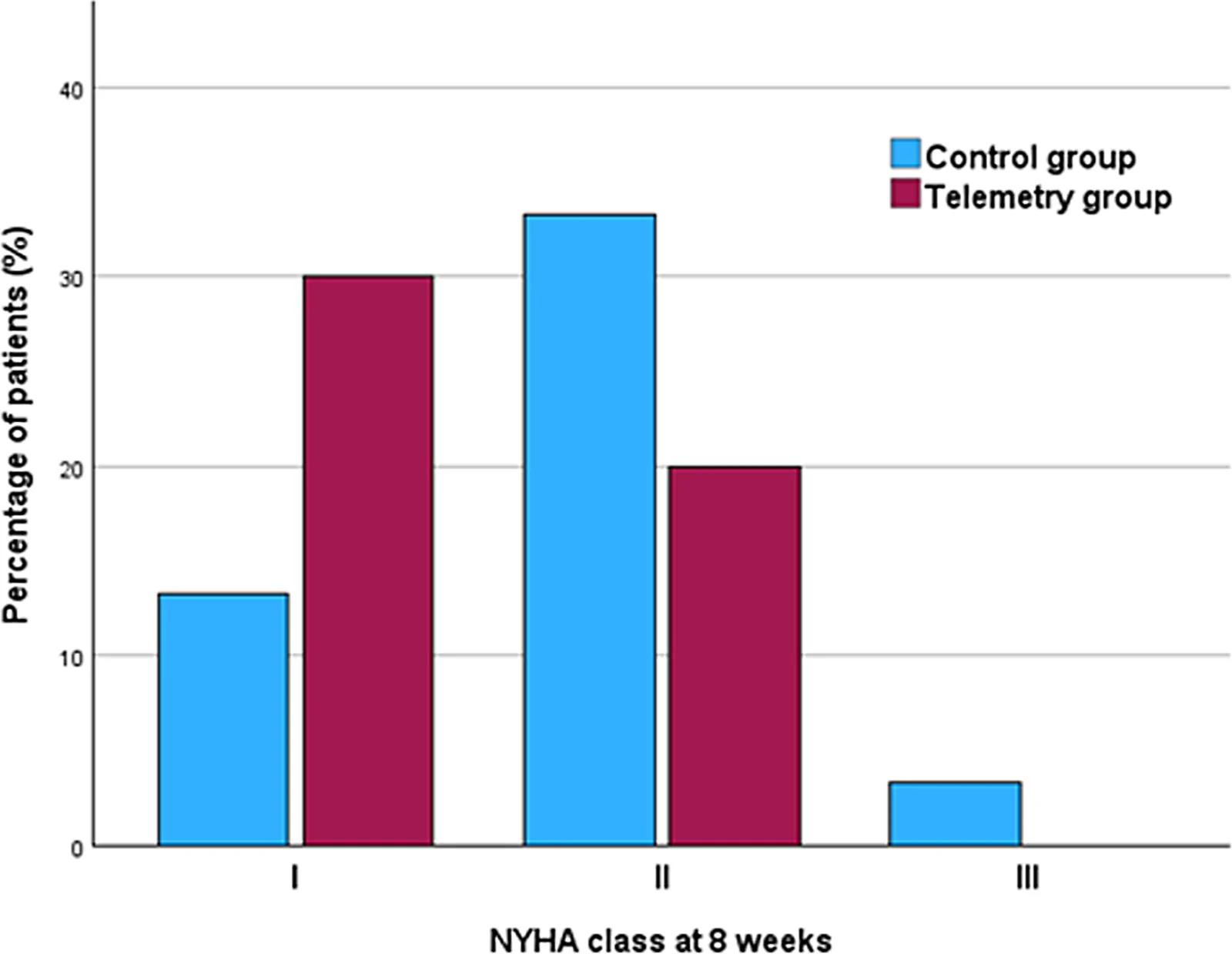
Distribution of NYHA functional class at 8 weeks according to study group. Following 8 weeks of follow-up, a greater proportion of patients in the telemetry group achieved NYHA functional class I, whereas most patients in the control group remained in NYHA class II.

**Fig. 6.**
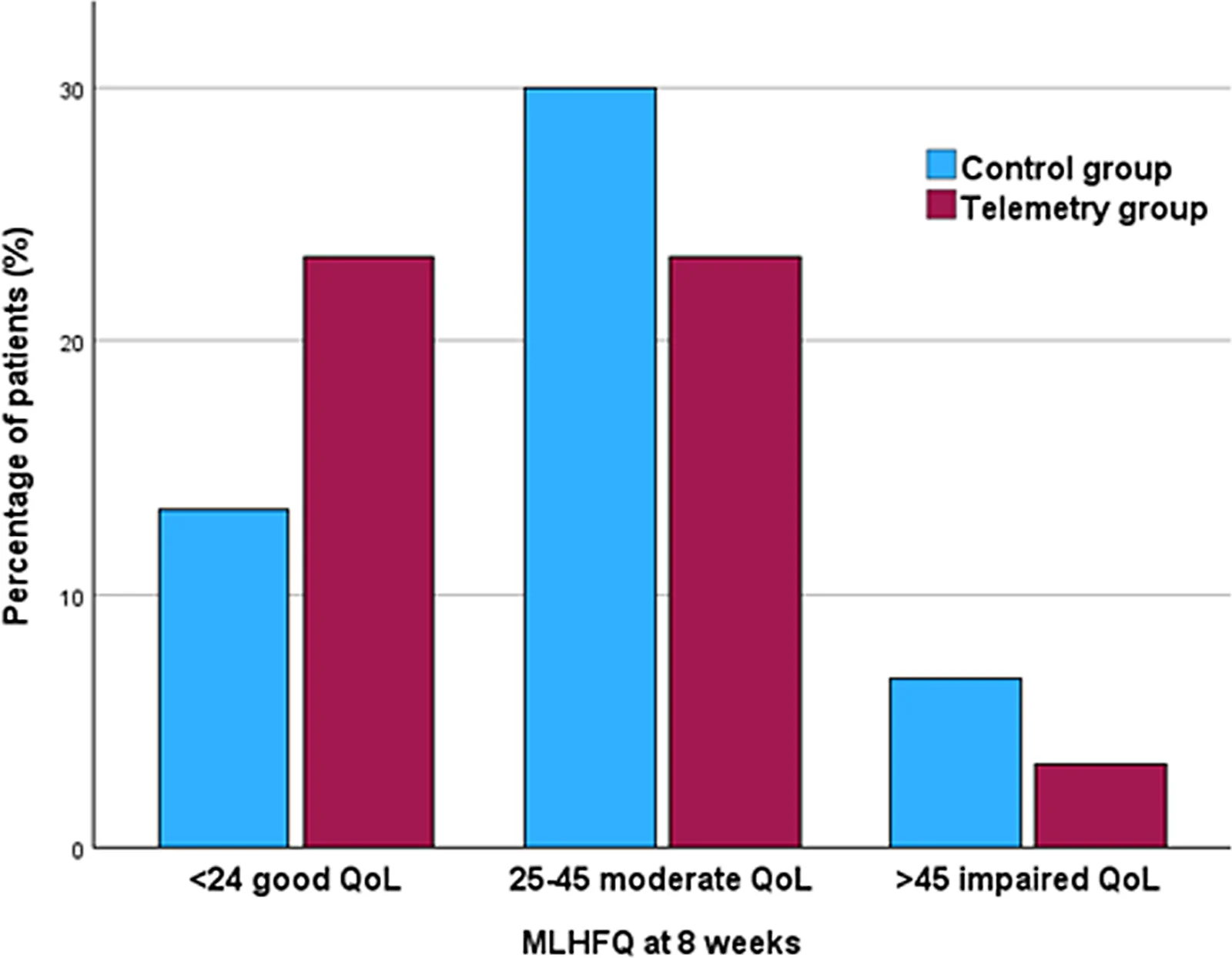
Distribution of MLHFQ categories at 8 weeks according to study group. After 8 weeks of follow-up, a greater proportion of patients in the telemetry group achieved good health-related QoL (MLHFQ <24 points), whereas moderate (25–45 points) and impaired (>45 points) QoL remained more frequent in the control group.

One patient in the telemetry group underwent primary prevention ICD implantation after continuous ambulatory monitoring identified recurrent monomorphic non-sustained ventricular tachycardia (Fig. 7, 8). The patient was a 36-year-old man with DCM and a family history of SCD. During hospitalization, repeat echocardiography confirmed persistently severe LV systolic dysfunction (LVEF 15%) despite optimization of GDMT. Following electrophysiology consultation and multidisciplinary clinical assessment, the combination of persistent severe LV dysfunction, recurrent ventricular arrhythmias, and an overall high arrhythmic risk supported the decision to proceed with primary prevention ICD implantation.

**Fig. 7.**
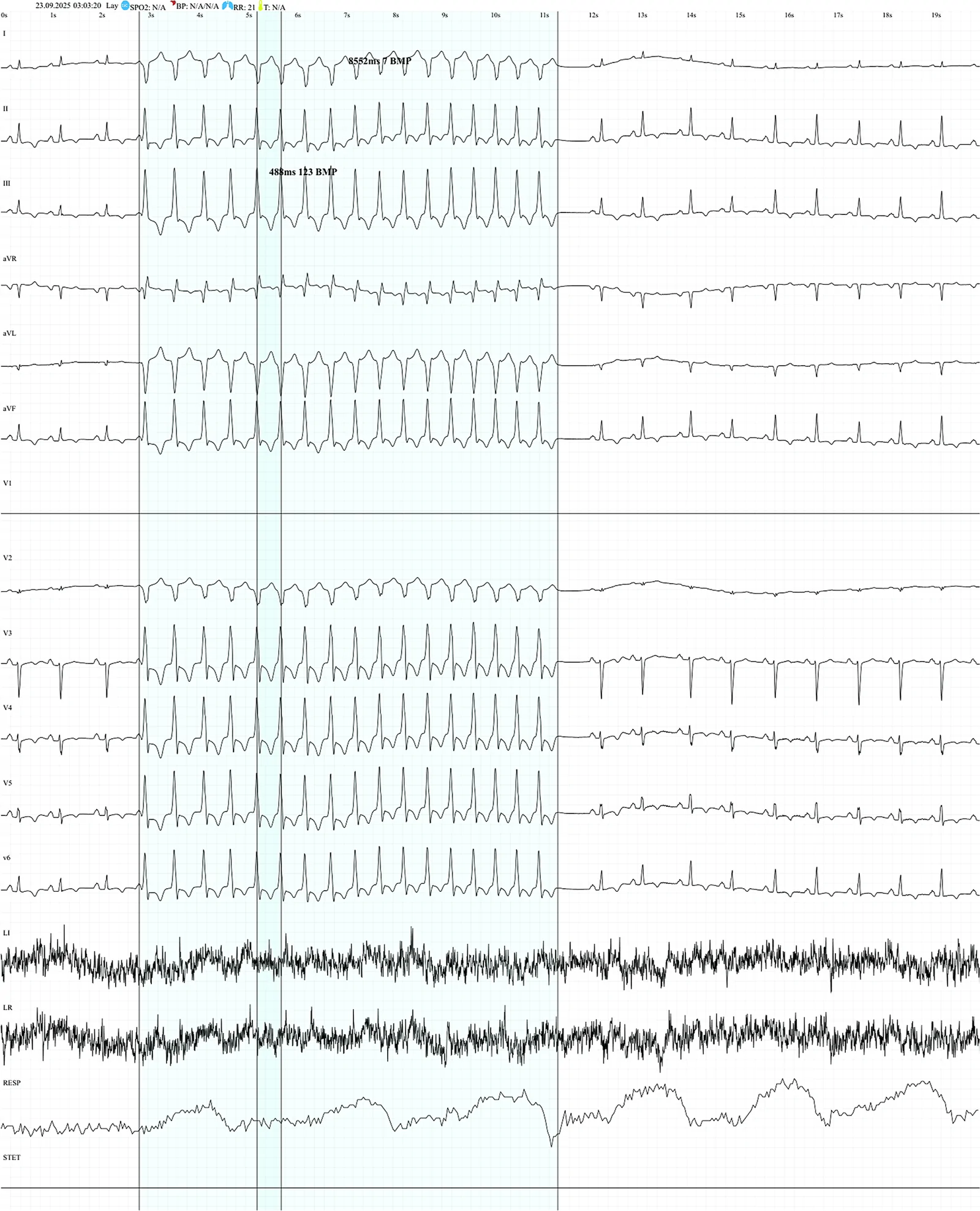
Representative telemetry recording showing non-sustained monomorphic VT with a ventricular rate of approximately 123 bpm (cycle length: 488 ms). The arrhythmia exhibited a regular wide QRS-complex morphology, spontaneous onset, and self-termination with restoration of sinus rhythm.

**Fig. 8.**
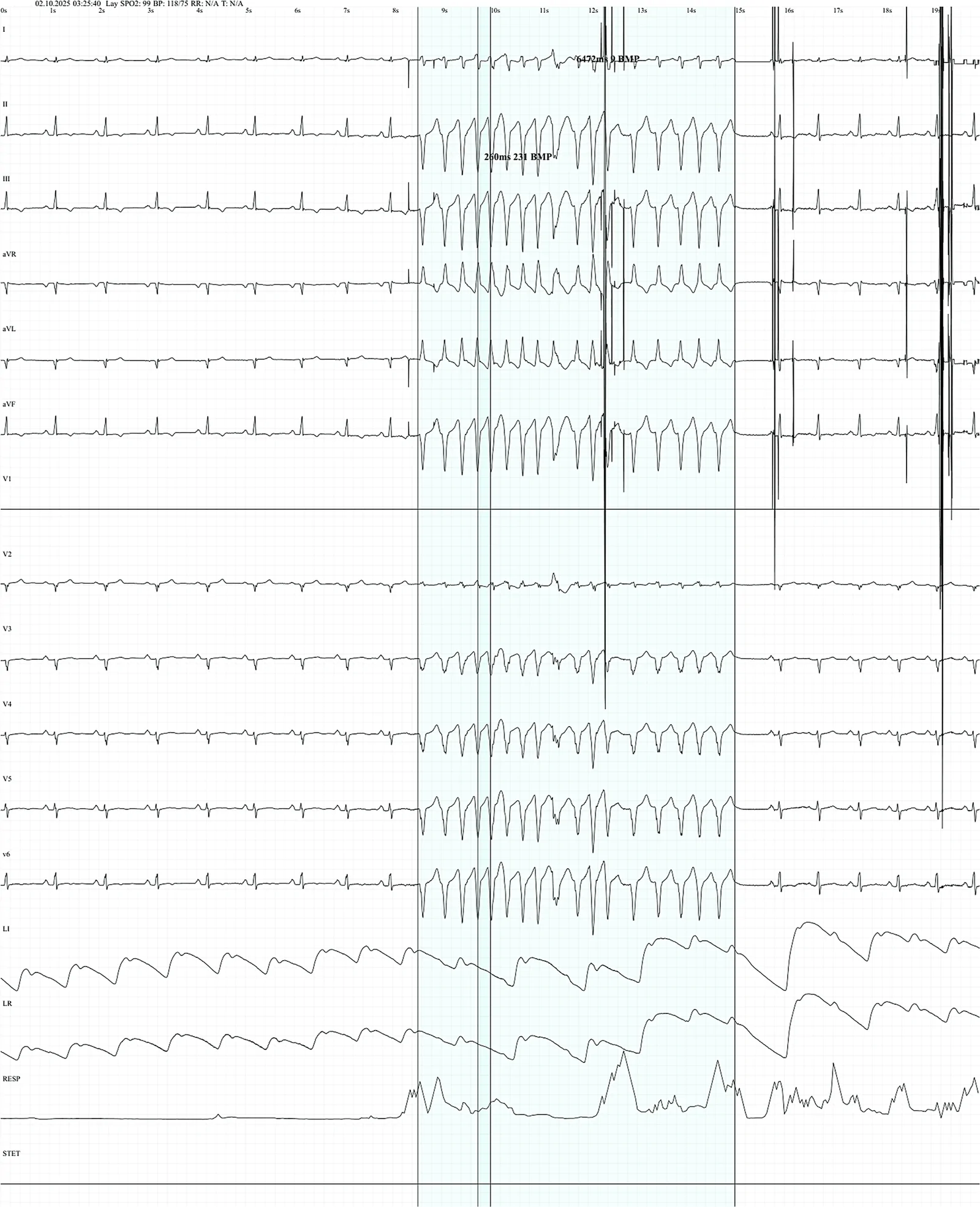
Representative telemetry recording showing rapid non-sustained monomorphic VT with a ventricular rate of approximately 231 bpm (cycle length: 260 ms). The episode was characterized by a regular wide QRS-complex rhythm with uniform morphology, abrupt onset, and spontaneous termination with restoration of sinus rhythm.

Across the entire telemetry monitoring period, follow-up events were observed predominantly in the telemetry group (Table 5). Unscheduled patient-initiated contacts occurred in 6 patients (40%) in the telemetry group, whereas no unscheduled calls were recorded in the control group, representing a statistically significant between-group difference (*p* = 0.017). Unscheduled outpatient visits were required in 4 telemetry patients (26.7%), while no such visits occurred in the control group; this difference did not reach statistical significance.

**Table 5.** Clinical events during the entire telemetry monitoring period

Parameters	Telemetry group (*n* = 15)	Control group (*n* = 15)	*p* value
Unscheduled calls, *n* (%)	6 (40.0)	0 (0)	0.017
Number of unscheduled visits, *n* (%)	4 (26.7)	0 (0)	0.100
Hospitalization due to arrhythmia, *n* (%)	4 (26.7)	0 (0)	0.100
ICD implantation, *n* (%)	1 (6.7)	0 (0)	1.000
Hospitalization due to HF, *n* (%)	0 (0)	0 (0)	NA
Death, *n* (%)	0 (0)	0 (0)	NA

ICD, implantable cardioverter-defibrillator; HF, heart failure; NA, not applicable.

As shown in Table 6, telemetry-guided management was associated with significantly greater achievement of the guideline-recommended BB TD throughout follow-up. The absolute between-group difference was 18.3% at weeks 1 and 2 (95% CI, 6.9%–29.7%; both *p* = 0.003), 16.7% at week 4 (95% CI, 6.0%–27.3%; *p* = 0.003), and 20.8% at week 8 (95% CI, 2.7%–39.2%; *p* = 0.028). ARNI target-dose achievement was consistently higher in the telemetry group, with absolute between-group differences ranging from 13.1% to 13.3%; however, the corresponding 95% CIs included zero, and none of these comparisons reached statistical significance. Similarly, MRA target-dose achievement was numerically higher in the telemetry group at all time points, with absolute differences ranging from 6.9% to 8.6%, although these differences were not statistically significant. Both groups achieved 100.0% of the SGLT2 inhibitor TD through week 4. At week 8, target-dose achievement was 96.7% in the telemetry group and 100.0% in the control group, yielding a nonsignificant absolute difference of −3.3% (95% CI, −10.4%–3.8%; *p* = 0.343).

**Table 6.** Absolute between-group differences in achievement of the guideline-recommended TD during follow-up according to GDMT medication class

Time point	Telemetry group	Control group	Absolute between-group difference (95% CI)	*p* value
ARNI				
Week 1	61.6%	48.3%	13.3 (−6.9–33.5)	0.187
Week 2	61.6%	48.3%	13.3 (−6.9–33.6)	0.187
Week 4	63.3%	50.0%	13.3 (−9.5–36.2)	0.242
Week 8	66.7%	53.6%	13.1 (−12.1–38.3)	0.295
BB				
Week 1	47.5%	29.2%	18.3 (6.9–29.7)	0.003
Week 2	47.5%	29.2%	18.3 (6.9–29.7)	0.003
Week 4	45.0%	28.3%	16.7 (6.0–27.3)	0.003
Week 8	60.0%	39.2%	20.8 (2.7–39.2)	0.028
MRA				
Week 1	53.6%	45.0%	8.6 (−0.5–17.6)	0.063
Week 2	53.6%	46.7%	6.9 (−1.9–15.7)	0.117
Week 4	53.8%	46.4%	7.4 (−2.1–16.9)	0.118
Week 8	51.8%	44.7%	7.1 (−3.1–17.4)	0.165
SGLT2 inhibitor				
Week 1	100.0%	100.0%	–	NA
Week 2	100.0%	100.0%	–	NA
Week 4	100.0%	100.0%	–	NA
Week 8	96.7%	100.0%	−3.3 (−10.4–3.8)	0.343

Data are presented as the mean percentage of the guideline-recommended TD achieved at each study visit. The absolute between-group difference is expressed as the telemetry group minus the control group, with corresponding 95% confidence intervals (CIs).

ARNi, angiotensin receptor-neprilysin inhibitor; BB, beta-blocker; CI, confidence interval; GDMT, guideline-directed medical therapy; MRA, mineralocorticoid receptor antagonist; SGLT2, sodium-glucose cotransporter-2; NA, not applicable.

Overall, the observed difference in GDMT optimization was predominantly attributable to more intensive BB up-titration in the telemetry group, whereas target-dose achievement for ARNI, MRA, and SGLT2 inhibitors was comparable between the groups. Given the exploratory nature of this pilot study, these findings should be interpreted cautiously but suggest that continuous remote monitoring may support earlier BB optimization while maintaining hemodynamic and laboratory stability.

## Discussion

The TELEMETRIC-HF pilot study evaluated the feasibility of integrating continuous multiparametric telemetry with clinician-led oversight during outpatient GDMT optimization in patients with HFrEF. The principal finding was that telemetry-guided management was associated with earlier and greater BB target-dose achievement, without evidence of clinically meaningful hemodynamic or biochemical deterioration. Continuous rhythm surveillance also identified ventricular arrhythmias that prompted additional clinical evaluation and intervention. These findings should nevertheless be interpreted cautiously. Although the study groups were generally comparable, ischemic cardiomyopathy was more prevalent in the control group, whereas the telemetry group had a numerically lower baseline LVEF and more previous HF hospitalizations. These differences were not statistically significant but may remain clinically relevant given the small sample size. The results should therefore be considered exploratory and hypothesis-generating.

The difference in GDMT optimization was predominantly attributable to BB up-titration. The telemetry group achieved a significantly greater proportion of the guideline-recommended BB TD throughout follow-up. The absolute between-group difference was 18.3% at weeks 1 and 2, 16.7% at week 4, and 20.8% at week 8, with statistically significant differences at all assessment points. In contrast, ARNI and MRA target-dose achievement was numerically higher in the telemetry group but did not differ significantly between groups. Both groups achieved 100% of the SGLT2 inhibitor TD through week 4, and no significant difference was observed at week 8. Thus, the observed effect of telemetry-guided care on pharmacological optimization appeared to be treatment-specific rather than reflecting uniformly greater intensification of all four foundational HFrEF therapies.

Slow GDMT titration remains an important implementation gap in routine HFrEF care and is frequently driven by concerns regarding hypotension, bradycardia, renal dysfunction, hyperkalemia, and clinical deterioration [16]. In TELEMETRIC-HF, greater BB exposure in the telemetry group was not accompanied by an excess of hypotension, worsening HF, or clinically meaningful biochemical instability. SBP and DBP remained clinically stable, and no HF-related hospitalizations occurred during follow-up. Continuous physiological surveillance and the capacity for prompt clinical reassessment may have reduced therapeutic inertia by providing clinicians with additional information regarding treatment tolerance between scheduled visits. However, because the study did not directly measure clinician confidence or therapeutic inertia, this mechanism remains inferential.

The present findings should be interpreted in the context of the STRONG-HF trial [21], which demonstrated that rapid GDMT optimization supported by frequent in-person visits, protocol-driven dose escalation, and serial clinical and biomarker assessment improved outcomes compared with usual care. Importantly, STRONG-HF achieved high rates of target-dose attainment without continuous telemetry, indicating that structured, high-frequency clinical contact is itself a major determinant of successful GDMT optimization. TELEMETRIC-HF was not designed to compare telemetry-guided care with the STRONG-HF strategy or to demonstrate that telemetry is superior to intensive clinical follow-up. Rather, it evaluated whether continuous ambulatory monitoring could feasibly complement outpatient care by providing physiological and rhythm surveillance during medication titration. Whether telemetry offers incremental benefit beyond an equally intensive protocol-driven follow-up strategy remains uncertain and requires direct evaluation in an adequately powered randomized trial.

Previous HF telemonitoring trials have yielded heterogeneous results, partly because the term “telemonitoring” encompasses interventions that differ substantially in monitoring intensity, clinical integration, and capacity to generate actionable information. TIM-HF2 demonstrated that structured remote patient management reduced the percentage of days lost because of unplanned cardiovascular hospitalization or all-cause death, supporting the value of a comprehensive telemedical strategy embedded within a defined clinical workflow [17]. In contrast, Tele-HF [22] and BEAT-HF [23], which relied primarily on symptoms and basic noninvasive measurements, did not significantly reduce readmissions or major clinical outcomes. Implant-based hemodynamic monitoring in CHAMPION reduced HF hospitalizations by linking continuously acquired physiological information to treatment modification [24]. Collectively, these studies suggest that clinical benefit depends less on remote data acquisition alone than on whether the collected data are clinically interpretable, reviewed within an effective organizational framework, and linked to timely therapeutic action.

Laboratory parameters remained broadly stable throughout the 8-week follow-up. Transient between-group differences in sodium and chloride concentrations were not accompanied by parallel evidence of clinical decompensation. The telemetry group had higher SBP during weeks 1–4 and a higher HR during weeks 4–8 despite receiving higher BB doses. These findings should not be interpreted as evidence of a harmful treatment effect. They may reflect differences in baseline autonomic tone, activity, clinical recovery, or other unmeasured characteristics and emphasize that BB titration should be based on overall clinical tolerance rather than HR alone. Unscheduled contacts in the telemetry group were predominantly related to rhythm findings rather than hypotensive symptoms, suggesting that the increased healthcare interaction was primarily driven by the detection of arrhythmic events.

Ventricular arrhythmias were identified only in the telemetry group, in which continuous rhythm monitoring was performed. Two patients had VT detected during the first week, and 3 patients had experienced VT cumulatively by the end of week 4. This finding is therefore inherently affected by surveillance and detection bias and cannot establish a higher underlying incidence of VT in the telemetry group. Intermittent clinic-based assessment may fail to identify transient arrhythmias in patients with HFrEF [25]; however, enhanced detection should not automatically be equated with clinical benefit. Arrhythmia-related hospitalization and initiation of antiarrhythmic therapy may represent appropriate responses to clinically important events, but they may also reflect a monitoring-induced diagnostic and therapeutic cascade. Because TELEMETRIC-HF was not powered to determine whether these interventions improved patient outcomes, the arrhythmia findings should be interpreted as evidence of diagnostic yield rather than evidence of reduced arrhythmic risk or prevention of SCD.

The higher frequency of arrhythmia-related hospitalization in the telemetry group should consequently receive a two-sided interpretation. Continuous rhythm surveillance enabled earlier specialist assessment and therapeutic intervention, but it also increased short-term healthcare utilization. Device-based remote monitoring studies have shown that frequent transmissions can identify clinically meaningful arrhythmias and physiological deterioration, and IN-TIME demonstrated improvements in clinical status and mortality with structured implant-based monitoring [25–27]. Nevertheless, the present study cannot determine whether the additional admissions and treatment changes prompted by telemetry were necessary, improved prognosis, or exposed patients to potentially avoidable interventions. Future studies should incorporate blinded arrhythmia adjudication, predefined thresholds for escalation of care, and outcome assessment to distinguish clinically beneficial intervention from surveillance-related overmanagement.

The ICD implantation performed during weeks 4–8 requires particularly cautious interpretation. Current ESC [19] and AHA/ACC/HFSA [20] guidelines generally recommend reassessment after an adequate period of optimized GDMT before primary-prevention ICD implantation in patients with reduced LVEF, allowing time for potential reverse remodeling. The 2021 ESC guideline recommends ICD implantation in patients with ischemic cardiomyopathy and persistent LVEF ≤35% despite ≥3 months of optimal medical therapy and states that ICD implantation should be considered in appropriately selected patients with nonischemic cardiomyopathy under similar conditions [19]. The DANISH trial [28] further demonstrated that prophylactic ICD implantation reduced SCD but did not significantly reduce all-cause mortality in patients with nonischemic systolic HF receiving contemporary therapy, reinforcing the need for individualized risk assessment.

In TELEMETRIC-HF, ICD implantation was not protocol-driven and was not based solely on the detection of VT. It followed multidisciplinary assessment of the overall clinical profile, including severe LV systolic dysfunction, recurrent ventricular arrhythmia, the underlying cardiomyopathy, and additional arrhythmic risk factors. This case should not be interpreted as evidence that telemetry can shorten or replace the guideline-recommended period of GDMT optimization. Rather, it illustrates the clinical uncertainty that may arise when potentially important ventricular arrhythmias are detected during this period. Whether such information should influence the timing of ICD implantation remains unresolved. In selected patients requiring temporary protection while awaiting reassessment after GDMT optimization, a wearable cardioverter-defibrillator may represent an alternative strategy because, unlike diagnostic telemetry, it is capable of detecting and terminating a malignant ventricular arrhythmia.

The findings of TELEMETRIC-HF should also be viewed within the broader field of mobile health and digitally supported cardiovascular care. Medical-grade telemetry primarily provides continuous physiological and rhythm surveillance, whereas smartphone applications and connected smart devices may offer complementary functions such as medication reminders, physical-activity tracking, patient education, individualized feedback, and remote coaching. Evidence from the LIGHT randomized clinical trial and its sub-studies suggests that digitally supported lifestyle interventions may favorably influence estimated atherosclerotic cardiovascular risk and functional capacity measured using peak oxygen consumption in individuals at high cardiovascular risk [29, 30]. Although these findings were obtained in a primary-prevention population and cannot be directly extrapolated to patients with HFrEF, they illustrate the potential value of combining physiological monitoring with interventions designed to improve engagement, adherence, and sustained behavioral change.

### Clinical Implications

Within the limitations of this pilot study, TELEMETRIC-HF supports the feasibility of incorporating continuous multiparametric telemetry into a structured outpatient pathway for patients undergoing GDMT optimization. Its principal potential clinical value lies in facilitating timely BB up-titration while allowing clinicians to assess physiological tolerance between scheduled visits. The platform used in the study had previously been evaluated for continuous vital-sign acquisition in high-risk patients in the NIGHTINGALE clinical validation study [31], supporting its use as a monitoring component within this exploratory care model.

The findings do not establish that telemetry prevents SCD, reduces arrhythmic risk, or enables earlier guideline-based ICD implantation. Instead, rhythm monitoring may provide supplementary information during the clinically uncertain period in which patients remain at reduced LVEF but have not yet completed the recommended duration of optimized medical therapy. Such information should complement, not replace, guideline-defined criteria, adequate GDMT exposure, reassessment of ventricular function, electrophysiological evaluation, and individualized consideration of temporary protection with a wearable cardioverter-defibrillator.

A comprehensive remote-management model could ultimately combine medical-grade rhythm and physiological monitoring with app-based education, medication-adherence support, activity monitoring, and structured clinician feedback. This model might address both clinician-level barriers to dose escalation and patient-level barriers to sustained treatment adherence. However, its clinical value will depend on clearly defined alert thresholds, standardized response protocols, adequate staffing, and evidence that increased surveillance improves patient-centered and cardiovascular outcomes without generating unnecessary interventions. Future multicenter trials should directly compare telemetry-supported care with equally intensive structured follow-up and determine whether the addition of continuous monitoring improves target-dose achievement, treatment persistence, ventricular remodeling, QoL, hospitalization, and survival.

### Study Limitations

Several limitations should be considered. First, the small sample size and single-center design limit statistical power, precision, and generalizability and increase the possibility that numerical baseline differences influenced the findings. Second, the 8-week follow-up was sufficient to examine early titration but was inadequate to assess sustained medication adherence, reverse remodeling, HF hospitalization, mortality, SCD, or the long-term consequences of telemetry-triggered interventions. Third, the open-label design and absence of allocation concealment introduced potential performance and selection bias. Fourth, continuous rhythm monitoring was performed only in the telemetry group, making detection bias unavoidable and preventing comparison of the true incidence of ventricular arrhythmias between groups. Finally, objective device-performance measures, including wear time and the proportion of analyzable data per patient-day, were not systematically recorded. These limitations reinforce the exploratory nature of the findings and the need for adequately powered multicenter studies with longer follow-up, standardized event adjudication, systematic documentation of titration barriers, and predefined clinical escalation pathways.

## Conclusions

In this small randomized pilot study, telemetry-guided remote monitoring was associated with faster BB up-titration and enhanced detection of clinically actionable ventricular arrhythmias during early HFrEF management. These findings support the feasibility of this approach and warrant confirmation in larger, adequately powered multicentre trials evaluating clinical outcomes.

## Statement of Ethics

The study was conducted in accordance with the Declaration of Helsinki and Good Clinical Practice guidelines. The study protocol was approved by the Institutional Ethics Committee of the National Institute for Heart and Blood Vessels “Dedinje” (the Approval No. 2446, dated March 25, 2023), and all participants provided written informed consent prior to enrollment.

## Conflict of Interest Statement

S.B. serves as a proctor for HeartMate 3 (Abbott) and SynCardia. D.K. has a professional service agreement with Abbott. All other authors have no conflicts of interest.

## Funding Sources

This work was supported by the E.U. under the R&D project No. 190185304 (Acronym: ARCHANGEL). The funder had no role in the design, data collection, data analysis, and reporting of this study.

## Author Contributions

Conceptualization, funding acquisition, methodology, investigation, and supervision: S.B.; project administration: S.B. and M.B.; data curation: S.B., J.M., B.M., G.L., J.L., and N.R.; formal analysis: N.R.; validation: S.B., D.K., J.L., J.M., G.L., J.S.N., B.M., P.D., N.R., and M.B.; writing – original draft and visualization: S.B. and N.R.; writing – review and editing: S.B., D.K., J.S.N., J.L., J.M., G.L., P.D., M.B., and N.R.; resources: S.B., D.K., J.S.N., J.M., P.D., and B.M. All authors participated in critically revising the manuscript for important intellectual content and approved the final version of the manuscript.

## Data Availability

The data supporting the findings of this study are available from the corresponding author upon reasonable request. Due to privacy and ethical considerations, the data are not publicly accessible.
